# Mental Health and Alcohol Consumption Among University Students in the Post-Pandemic Context: An Exploratory Cross-Sectional Study in Portugal

**DOI:** 10.3390/healthcare14020223

**Published:** 2026-01-16

**Authors:** Maria Teresa Moreira, Maria Inês Guimarães, Augusta Silveira, Beatriz Loibl, Beatriz Guedes, Hugo Ferraz, Inês Castro, Sofia Mira de Almeida, Inês Lopes Cardoso, Sandra Rodrigues, Andreia Lima

**Affiliations:** 1 Research Unit in Health (RISE-Health), Fernando Pessoa School of Health, Fernando Pessoa–Institute for Research, Innovation and Development (FP-I3ID), Fernando Pessoa Education and Culture Foundation, Rua Delfim Maia 334, 4200-253 Porto, Portugal; sandrar@ufp.edu.pt; 2 Research Unit in Health (RISE-Health), Faculty of Health Sciences, Fernando Pessoa University, Fernando Pessoa–Institute for Research, Innovation and Development (FP-I3ID), Fernando Pessoa Biomedical and Health Sciences (FP-BHS), Fernando Pessoa Education and Culture Foundation, Rua Carlos da Maia 296, 4200-150 Porto, Portugal; inesg@ufp.edu.pt (M.I.G.); augusta@ufp.edu.pt (A.S.); mic@ufp.edu.pt (I.L.C.); 3 North Delegation—National Institute of Legal Medicine and Forensic Sciences (INMLCF), Jardim Carrilho Videira, 4050-167 Porto, Portugal; 4 Centre of Investigation in Technologies and Centre for Health Studies and Research of the University of Coimbra (CEISUC), 3004-512 Coimbra, Portugal; 5 Faculty of Health Sciences, Fernando Pessoa University (FCS-UFP), 4240-004 Porto, Portugal; 42806@ufp.edu.pt (B.L.); 42416@ufp.edu.pt (B.G.); 42439@ufp.edu.pt (H.F.); 39700@ufp.edu.pt (I.C.); 6 Egas Moniz Faculty, Sul Delegation—National Institute of Legal Medicine and Forensic Sciences, Jardim Carrilho Videira, 4050-167 Porto, Portugal; 7 Escola de Medicina e Ciências Biomédicas (EMCB), Fundação Ensino e Cultura Fernando Pessoa (FFP), Avenida Fernando Pessoa 150, 4420-096 Gondomar, Portugal; 8 The Health Sciences Research Unit of Nursing, Escola Superior de Saúde–Instituto Politécnico de Viana do Castelo (ESS-IPVC), 4900-314 Viana do Castelo, Portugal; amarialima@ess.ipvc.pt

**Keywords:** alcohol use, university students, depression, anxiety, psychological stress, COVID-19

## Abstract

**Introduction:** The COVID-19 pandemic had significant effects on mental health and lifestyle behaviours, especially among university students who experienced academic disruptions, social isolation, and fewer social interactions. Alcohol consumption has long been part of student culture. Still, the influence of post-pandemic academic reintegration on drinking patterns and psychological distress remains relatively unexplored, particularly in countries like Portugal, where student traditions heavily shape consumption habits. This study aimed to describe the prevalence of alcohol consumption, depression, anxiety, and stress in a sample of Portuguese university students during the post-pandemic academic period, and to explore associations with sociodemographic variables. **Methods:** A cross-sectional study was conducted in November 2021 with 90 students from a private higher education institution in northern Portugal. Data were collected via an online questionnaire including sociodemographic information, the Alcohol Use Disorders Identification Test (AUDIT), and the Depression, Anxiety and Stress Scale (DASS-21). **Result:** The majority of the participants were not at risk of alcohol addiction (95.3%). In total, 15.1% of students reported anxiety symptoms ranging from severe to extremely severe. A binomial logistic regression was performed to ascertain the effects of being away from home and psychological distress (DASS-42 score), on the likelihood that participants were at risk of alcohol addiction (Level 3 and 4 in the AUDIT scale). The logistic regression model was statistically significant, χ^2^(2) = 9.20, *p* = 0.010. Living away from home was associated with a substantially lower likelihood of high-risk status (*B* = −2.79, *p* = 0.034), corresponding to an odds ratio of 0.06, indicating a strong protective effect. DASS-42 total score was positively associated with high-risk status (*B* = 0.04, *p* = 0.039), such that higher psychological distress increased the odds of being classified as high risk. **Conclusions:** The findings reveal a low prevalence of alcohol risk but heightened symptoms of anxiety, depression, and stress. Psychological distress notably increases the likelihood of hazardous alcohol use, emphasising the importance of targeted mental health and alcohol-use interventions among university students.

## 1. Introduction

The COVID-19 pandemic profoundly disrupted the lives of university students, a population already recognised as vulnerable to psychological distress. Prolonged periods of social isolation, abrupt transitions to online learning, academic uncertainty, and reduced peer interaction contributed to increased levels of anxiety, depression, and stress during lockdowns [1,2,3,4]. Importantly, emerging evidence suggests that these psychological effects did not fully resolve with the easing of restrictions, but persisted into the post-pandemic phase, particularly during the period of academic reintegration [2,4]. This transitional stage represents a critical moment for understanding students’ emotional well-being and associated health behaviours [5,6].

Recent evidence indicates that the consequences of COVID-19 in young adults extend beyond the acute phase of infection and the period of strict public-health restrictions. University students continue to report persistent symptoms, including fatigue, emotional dysregulation, reduced physical functioning, and changes in sleep and daily routines, even after in-person academic activities resumed [7,8]. These post-COVID sequelae are known to interfere with lifestyle behaviours, including physical activity, diet, and substance use, potentially increasing vulnerability to maladaptive coping strategies such as alcohol consumption [2,3]. Large-scale, longitudinal studies in university populations have shown that the pandemic and its aftermath were associated with significant changes in drinking patterns, mental well-being, and health-related behaviours, reflecting the complex interaction between lingering physical and psychological burden and social reintegration [1,7,8]. Framing alcohol use in the post-pandemic period therefore requires acknowledging not only social and emotional stressors but also the persistent health effects of COVID-19 that continue to shape students’ daily functioning and behavioural choices.

Alcohol consumption has long been part of university social life and remains one of the most common risk behaviours among students. International research repeatedly shows that university environments encourage permissive drinking norms, increasing both the frequency of alcohol use and binge drinking compared to non-student peers [6,9]. Before the pandemic, a significant number of university students already met criteria for anxiety or mood disorders, with alcohol misuse often acting as a maladaptive coping mechanism [1,7]. During the pandemic, however, patterns of alcohol consumption became more varied. While some students reported drinking more in response to stress, loneliness, and emotional distress, others decreased their intake due to limited access to social venues, financial difficulties, or increased family supervision [1,3,7]. These different paths illustrate the complexity of alcohol-use behaviours during times of social upheaval and recovery.

The post-pandemic academic period could be a particularly delicate phase, as students return to in-person teaching, social interactions, and traditional academic celebrations while still experiencing residual psychological distress. Theoretical frameworks such as the Tension Reduction Hypothesis and the Drinking Motives Model offer a valuable perspective for understanding these dynamics, indicating that alcohol might be used both to reduce negative feelings and to support social reintegration [3,7]. In this context, students may simultaneously partake in socially motivated drinking and rely on alcohol as a coping strategy, increasing their risk of hazardous consumption patterns.

In Portugal, these dynamics should be understood within a unique cultural and academic setting. Portuguese university life is heavily influenced by collective rituals and traditions, such as praxe (initiation rituals) and major academic celebrations like Queima das Fitas, which are closely linked to heavy alcohol consumption and binge drinking [6,10]. Previous national research has documented concerning levels of alcohol use among Portuguese university students, especially during academic festivities [10]. Despite this, there is limited empirical evidence exploring the connection between mental health and alcohol consumption in the post-pandemic era. Moreover, much of the existing literature on student alcohol use during COVID-19 has concentrated on Northern Europe and North America, leaving Southern European contexts, including Portugal, underrepresented [2,3].

Understanding alcohol-use behaviours within this cultural framework is especially important given the relatively small-scale nature of many institutional studies conducted in Portugal. In such contexts, the value of research lies not in broad generalisability but in its ability to provide culturally grounded insights that reflect local academic practices and social norms. Studying Portuguese students during the early stages of post-pandemic academic reintegration offers an opportunity to explore how psychological distress and alcohol consumption intersect within a setting where alcohol plays a central role in student socialisation.

Therefore, this study aimed to examine the relationship between alcohol consumption and psychological distress (depression, anxiety, and stress) among Portuguese university students during the post-pandemic academic period. Additionally, it explored how sociodemographic factors, including living arrangements, shape alcohol-use risk in this cultural context. We hypothesised that higher levels of psychological distress would be associated with an increased likelihood of risky alcohol consumption and that living away from home would influence students’ alcohol-use patterns.

## 2. Materials and Methods

### 2.1. Study Design and Participants

This study used an observational, cross-sectional design, suitable for exploring links between psychological distress and alcohol use at a specific moment, especially in quickly changing public health situations like the post-COVID-19 period [1,2]. A non-probabilistic convenience sampling method was employed, reflecting the exploratory nature of the research and the limited size of the institutional population involved.

Participants were recruited from a private higher education institution located in northern Portugal. Inclusion criteria were enrolment at the institution and being at least 18 years old. At the time of data collection, the total student population was approximately 100 students. To ensure adequate statistical precision, the minimum required sample size was estimated using an online sample size calculator (Raosoft^®^, Seattle, WA, USA), assuming a 95% confidence level, a 5% margin of error, and a conservative response distribution of 50%. Based on these parameters, at least 80 participants were needed. Data collection was completed once this target was reached, resulting in a final sample of 90 students.

Although the sample size limits generalisability, it is sufficient for exploratory analysis within a clearly defined cultural and institutional context, consistent with previous studies examining student mental health and alcohol consumption in Portuguese higher education settings [7,10].

### 2.2. Data Collection Procedure

Data collection was conducted in November 2021, during a transitional phase characterised by high vaccination coverage and the gradual resumption of in-person academic activities in Portugal. At this time, social distancing restrictions had been largely lifted, and university life—including initiation rituals (praxe) and academic festivities traditionally associated with alcohol consumption—was progressively reinstated. This timing is particularly relevant, as it captures student behaviours during early post-pandemic academic reintegration, a period identified as critical for mental health outcomes and risk behaviours [1,3].

Participants were invited to participate via institutional email and completed a self-administered online questionnaire hosted on Google Forms. Online data collection was selected to facilitate accessibility and ensure participant anonymity, which has been shown to improve the reliability of self-reported data on sensitive behaviours such as alcohol consumption and psychological symptoms [3,9]. Before accessing the questionnaire, participants were provided with detailed study information and an electronic informed consent form. Only students who provided consent were permitted to proceed.

### 2.3. Instruments

#### 2.3.1. Sociodemographic and Academic Questionnaire

A structured questionnaire was used to collect sociodemographic and academic information, including age, sex, nationality, marital status, degree programme, year of study, working student status, and living arrangements. Participants were asked whether they had changed residence upon entering higher education and whether they were currently living away from home. Living arrangements were included as a key variable given their documented association with student adjustment, mental health, and alcohol consumption patterns [6,7].

#### 2.3.2. Alcohol Use Disorders Identification Test (AUDIT)

Alcohol consumption was assessed using the Alcohol Use Disorders Identification Test (AUDIT), a 10-item screening instrument developed by the World Health Organization to identify hazardous and harmful alcohol use across diverse populations [11]. The AUDIT evaluates three domains: alcohol consumption, drinking behaviour, and alcohol-related problems. The Portuguese validated version of the instrument was used and demonstrated good psychometric properties in university populations [6].

Total AUDIT scores range from 0 to 40 and are classified into four risk zones: low risk or abstinence (0–7), moderate risk (8–15), harmful use (16–19), and probable dependence (≥20), in accordance with WHO guidelines [11]. For inferential analysis, participants were dichotomised into low-risk (zones I and II) and high-risk (zones III and IV) groups. This approach has been commonly used in previous research to enhance statistical power when examining predictors of alcohol-use risk among university students [3,7].

#### 2.3.3. Depression, Anxiety and Stress Scale (DASS-21)

Psychological distress was assessed using the Depression, Anxiety and Stress Scale-21 items (DASS-21), a widely used self-report instrument that measures core symptoms of depression, anxiety, and stress experienced over the previous week [12]. Each subscale contains seven items rated on a 4-point Likert scale, with higher scores indicating greater symptom severity.

Severity thresholds were applied using established cut-off values for the Portuguese population [12]. To be comparable with studies using the original 42-item version, subscale scores were summed and multiplied by two, yielding a total DASS-42 score. This total score was used as a continuous variable in the regression analysis to represent overall psychological distress, consistent with previous international and national research examining mental health and substance use among university students [2,3,9].

### 2.4. Statistical Analysis

Statistical analyses were performed using the Statistical Package for the Social Sciences (SPSS), version 30.0. Descriptive statistics were computed to summarise sociodemographic characteristics, alcohol consumption patterns, and psychological distress levels. Continuous variables were reported as means and standard deviations, while categorical variables were described using frequencies and percentages.

To examine factors associated with alcohol-use risk, a binomial logistic regression analysis was conducted. Alcohol-use risk, dichotomised based on AUDIT categories, served as the dependent variable. Independent variables were selected a priori based on theoretical relevance and empirical evidence, and included living away from home and psychological distress (total DASS-42 score) [1,3,7]. The model was estimated using the Enter method. Model fit was evaluated using the omnibus test of model coefficients, pseudo-R^2^ indices (Cox & Snell; Nagelkerke), and the Hosmer–Lemeshow goodness-of-fit test. Results were expressed as odds ratios with 95% confidence intervals. Statistical significance was set at *p* ≤ 0.05.

### 2.5. Ethical Considerations

This study was conducted in accordance with the principles of the Declaration of Helsinki. Ethical approval was obtained from the Ethics Committee of the University Fernando Pessoa (FCS/PI-3620, approved on 10 March 2020). All participants provided informed consent before participation. Data were collected anonymously, stored securely, and used exclusively for research purposes, ensuring confidentiality and compliance with ethical standards.

## 3. Results

The sample included 90 participants, aged 23.64 ± 7.00, ranging from 18 to 51 years. Most were female (82.2%), single (86,7%), Portuguese (85.6%), in their first year of the degree (52.2%), and were not away from home (73.3%), with 60% of these living with their parents. Regarding their degree programmes, 43.3% of the students were enrolled in nursing. Among those 26.6% who had changed residence, 45.8% lived with colleagues. Within the sample, 17.4% identified as working students, as shown in Table 1.

As shown in Table 2, most students (86.0%) fall within the low risk and abstinence zone; however, 9.3% were classified in moderate risk, 3.5 in harmful use and 1.2 in probable dependence.

As evidenced in Table 3, 15.1% of students reported anxiety symptoms ranging from severe to extremely severe. For all subscales, level I (normal scores) recorded the highest percentage. However, level 3 symptoms (moderate) are still very prevalent for all subscales.

A binomial logistic regression was performed to ascertain the effects of being away from home and the score on the DASS scale, on the likelihood that participants were at risk of alcohol addiction (Level 3 and 4 in the AUDIT scale). The logistic regression model (Table 4) was statistically significant, χ^2^(2) = 9.20, *p* = 0.010, indicating that the set of predictors reliably distinguished between individuals with and without high-risk status. The model accounted for 10.3% of the variance according to Cox and Snell R^2^ and 32.5% according to Nagelkerke R^2^.

The Hosmer–Lemeshow test was non-significant, χ^2^(7) = 3.30, *p* = 0.856, suggesting an adequate model fit. Overall classification accuracy was 96.5%, although this value should be interpreted with caution due to the low prevalence of high-risk cases.

Both predictors were statistically significant. Living away from home was associated with a substantially lower likelihood of high-risk status (*B* = −2.79, *p* = 0.034), corresponding to an odds ratio of 0.06, indicating a strong protective effect. DASS-42 total score was positively associated with high-risk status (*B* = 0.04, *p* = 0.039), such that higher psychological distress increased the odds of being classified as high risk.

## 4. Discussion

This study offers insight into the complex relationship between psychological distress and alcohol consumption among Portuguese university students during the post-pandemic academic period. Although most participants were classified as low-risk drinkers, a significant proportion had moderate to high AUDIT scores, indicating that hazardous alcohol use persists in specific subgroups. At the same time, a considerable number of students reported moderate to severe symptoms of anxiety, depression, and stress, suggesting that the psychological impact of the COVID-19 pandemic extended beyond the period of strict restrictions [1,2,3,13,14,15,16]. This persistence aligns with emerging evidence of post-COVID symptoms in young adults, including fatigue and reduced physical functioning, which may further contribute to emotional distress and maladaptive coping behaviours such as alcohol use.

The association between higher DASS-42 scores and an increased likelihood of alcohol-use risk aligns with a growing body of international research emphasising the bidirectional relationship between mental health issues and substance use among university students [2,3,7,8]. Anxiety, depression, and stress have consistently been recognised as key factors influencing alcohol misuse, particularly during periods of prolonged uncertainty and upheaval, such as the COVID-19 pandemic [1,13,14,15,16,17].

In the Portuguese academic setting, the relatively low prevalence of risky alcohol consumption observed in this study should be interpreted with caution. Pre-pandemic national studies reported high levels of alcohol use and binge drinking among Portuguese university students, particularly during academic celebrations and initiation rituals [6,10]. The lower levels found in the current sample may reflect behavioural changes developed during the pandemic, such as increased health consciousness, altered social routines, or reduced tolerance for excessive drinking following prolonged disruption [1,18].

However, the ongoing prevalence of moderate to high-risk drinking among some students shows that alcohol-related harm remains an important concern. In Portugal, alcohol is a key part of academic socialisation, with traditions such as praxe and Queima das Fitas serving as powerful normative contexts for drinking [6,10]. These settings can increase students’ vulnerability to psychological distress, helping shift socially normal drinking towards risky patterns. Similar rebounds in alcohol consumption after lockdown, as social activities resumed, have been observed in other Southern European contexts [18,19], highlighting the importance of culturally relevant interpretations.

Instead of indicating a sustained reduction in risk, the findings might signal a transitional phase in which alcohol consumption patterns are still stabilising. As academic traditions and social events fully resume, students experiencing unresolved emotional distress may face heightened social pressures centred on alcohol.

One of the most intriguing findings of this study is the protective effect linked to living away from home. Contrary to pre-pandemic literature, which often identifies relocation as a risk factor for increased alcohol consumption among university students [6,9], our results indicate that, in the post-pandemic context, living away from home may be associated with a lower risk of alcohol use.

This finding may indicate greater autonomy, self-regulation, and adaptive coping among students who successfully transitioned to independent living during a period of significant disruption. Living away from home might have helped develop structured routines, peer support networks, and adaptive coping strategies that lowered reliance on alcohol for emotional regulation [1,8]. Conversely, students living with family may have faced different stressors, such as reduced autonomy, role conflicts, or difficulties reintegrating socially, which could increase psychological distress during the post-pandemic transition [4,18].

These results highlight the importance of analysing sociodemographic predictors within their specific time and social context. Post-pandemic student groups may differ significantly from pre-pandemic populations, highlighting the need to revisit previously established risk profiles in light of unprecedented social upheaval.

The findings of this study have significant implications for higher education institutions, particularly in the Portuguese context. The strong link between psychological distress and alcohol-use risk underscores the need for integrated prevention strategies that address mental health and substance use simultaneously [3,8]. Screening for anxiety, depression, and stress may serve as an effective first step in identifying students at higher risk of hazardous drinking.

Universities and student associations play a crucial role in shaping students’ health behaviours. While they can promote social integration and peer support, they may also unintentionally reinforce risky drinking norms through alcohol-centric academic traditions [6,10]. Prevention efforts should therefore focus on encouraging alternative forms of socialisation, enhancing mental health literacy, and challenging the normalisation of binge drinking, especially during high-risk academic events.

Evidence suggests that structured psychological interventions, including cognitive-behavioural and psychosocial approaches delivered in academic settings, can effectively reduce emotional distress and related risk behaviours among students [13,19,20,21,22,23,24]. In the post-pandemic period, such interventions may be particularly beneficial in supporting students through periods of academic and social readjustment.

Several limitations should be considered when interpreting these findings. The cross-sectional design limits causal inference, and the small convenience sample limits generalisability. The limited number of participants in the highest AUDIT risk categories may have reduced the accuracy of regression estimates, and reliance on self-reported measures introduces potential reporting bias.

On the other hand, this study provides empirically supported evidence from a Southern European context that remains underrepresented in the literature, particularly regarding post-pandemic student behaviour [2,3]. By explicitly situating the analysis within the Portuguese academic and cultural framework, the study offers context-aware insights that complement larger international investigations.

Future research should use longitudinal designs to examine trajectories of psychological distress and alcohol consumption across the stages of academic reintegration [2,3,7,8,25,26,27,28,29]. Multicentre studies across Portuguese universities would improve representativeness and enable comparisons between institutions with diverse academic cultures. Qualitative methods could further clarify students’ lived experiences, coping strategies, and perceptions of academic drinking norms. Lastly, assessing the effectiveness of culturally adapted mental health and alcohol-prevention programmes will be crucial for guiding evidence-based policy and practice in higher education [21,30].

## 5. Conclusions

This study examined the relationship between alcohol consumption and psychological distress among Portuguese university students during the post-pandemic academic period, a critical phase marked by the gradual resumption of academic and social life after prolonged disruption. The findings indicate that, although the overall prevalence of hazardous alcohol consumption was low, a meaningful subgroup of students exhibited moderate to high alcohol-use risk. At the same time, symptoms of anxiety, depression, and stress remained prevalent, confirming that the psychological consequences of the COVID-19 pandemic persisted beyond the lifting of restrictive measures.

Psychological distress emerged as a central factor associated with alcohol-use risk. Higher overall distress was significantly associated with an increased likelihood of belonging to harmful or dependent AUDIT categories, reinforcing the notion that alcohol consumption among university students cannot be interpreted solely as a social behaviour but must also be understood in relation to emotional vulnerability and coping processes. This finding is particularly relevant in the post-pandemic context, where social drinking opportunities have resumed while psychological recovery appears uneven across individuals.

An important and unexpected finding was the protective role of living away from home. Contrary to pre-pandemic evidence that often identifies relocation as a risk factor for alcohol misuse, the present findings suggest that, during post-pandemic academic reintegration, living away from home may be associated with greater autonomy, adaptive coping, and reduced reliance on alcohol as a regulatory strategy. This highlights the importance of reassessing previously established risk profiles in light of the profound social and behavioural changes induced by the pandemic.

Interpreted within the Portuguese academic context, these results take on particular significance. Academic traditions and collective rituals, such as praxe and large-scale festivities, remain central to student socialisation and are closely intertwined with alcohol consumption. Even when overall risk levels are relatively low, students experiencing psychological distress may be especially vulnerable to alcohol-related harm in these normative environments. Therefore, the post-pandemic period should be viewed as a transitional and potentially sensitive phase, rather than a simple return to pre-pandemic patterns.

From an applied perspective, the findings underscore the need for integrated approaches to student health in higher education. Mental health promotion and alcohol-use prevention should not be treated as separate domains but as interrelated dimensions of student well-being. Early identification of psychological distress, combined with culturally sensitive prevention strategies that take into account the specificities of Portuguese academic life, may help reduce alcohol-related risks during periods of social reintegration.

Although limited by its cross-sectional design, small convenience sample, and reliance on self-reported measures, this study provides valuable context-specific evidence from a Southern European setting that remains underrepresented in the literature. By situating alcohol consumption within its psychological and cultural context, the study adds nuance to international findings and supports the development of targeted, culturally adapted interventions.

Future research should prioritise longitudinal and multicentre designs to clarify causal pathways and monitor changes in mental health and alcohol-use behaviours over time. Evaluating the effectiveness of integrated mental health and alcohol-prevention programmes will be essential to strengthening student resilience and promoting healthier academic environments in the post-pandemic era.

## Figures and Tables

**Table 1:** Sociodemographic characteristics of the university students.

Sociodemographic Characteristics		Sample (*n* = 90)		
		*n*		(%)
Sex	Female	74		82.2
	Male	16		17.8
Marital state	Single	78		86.7
	Married/Living with partner	12		13.3
	Divorced/Separated	0		0.0
Nationality	Portuguese	77		85.6
	Other	13		14.4
Degree Program	Nursing	39		43.3
	Dentistry	20		22.2
	Other	31		34.5
Year of degree	1º year Degree	47		52.2
	2º year Degree	13		14.4
	3º year Degree	14		15.6
	Other	16		17.8
Did you change your residence when you entered higher education?	Yes	24		26.6
	No	66		73.3
If yes, where do you live? (*n* = 24)	student residence	5		20.8
	Alone	3		12.5
	With colleagues	11		45.8
	With partner	3		12.5
	Other living arrangement	2		8.3
Do you have working student status?	Yes	15		17.4
	No	71		82.6
	Mean	SD	MIN	MAX
Age	23.6	7.0	18	51

Note: *n* = total sample; % = percentage; SD = standard error; MIN = Minimum; MAX = Maximum.

**Table 2:** Risk categories of the AUDIT scale.

AUDIT—Risk Levels Sample: *n* = 90				
	Zone I	Zone II	Zone III	Zone IV
Per (%)	86.0	9.3	3.5	1.2
AUDIT (Mean ± SD) 3.50 ± 4.61				

Note: Zone I: Low risk and abstinence, Zone II: Moderate risk, Zone III: Harmful use, Zone IV: Probable dependence.

**Table 3:** Subscale Scores of the Questionnaire DASS-21. Mean and standard deviation of the DASS-42 scale were reported to match the original scale.

	Distribution of Students by Severity Levels on the DASS-21 Scale (%)		
Sample (*n* = 90)	Depression	Anxiety	Stress
Level I	63.0	60.3	76.4
Level II	19.2	6.8	13.9
Level III	15.1	17.8	9.7
Level IV	2.7	13.7	-
Level V	-	1.4	-
DASS-42 (Mean ± SD): 47.89 ± 28.48			

Note: Level I: Normal score, Level II: Mild symptoms, Level III: Moderate symptoms, Level IV: Severe symptoms, Level V: Extremely severe symptoms. For the final score, the values of each subscale were added together and multiplied by two to match the score of the original scale (DASS-42).

**Table 4:** Results of the binary logistic regression predicting [dependent variable].

Predictor	B	SE	Wald	df	*p*	OR	OR 95% CI
Living away f/home	−2.786	1.317	4.477	1	0.034	0.06	[0.01, 0.81]
DASS-42	0.038	0.018	4.253	1	0.039	1.04	[1.00, 1.08]
Constant	−1.041	1.842	0.320	1	0.572	0.35	—

Note. B = logistic regression coefficient; SE = standard error; OR = odds ratio; CI = confidence interval. The model was estimated using binary logistic regression (Enter method). The dependent variable was coded as 0 = no high risk and 1 = high risk.

## Data Availability

The datasets presented in this article are not readily available because of confidentiality and ethical restrictions related to the protection of participants’ personal and health information, as required by the study’s ethics approval and informed consent agreements. However, anonymized data may be made available from the corresponding author upon reasonable request and subject to approval by the institutional ethics committee.

## Abbreviations

The following abbreviations are used in this manuscript:

AUDIT	Alcohol Use Disorders Identification Test
COVID-19	Coronavirus 2019
DASS	Depression Anxiety Stress Scales
EADS 21	Escala de Ansiedade, Depressão e Stress EADS-21
OMS	Organização Mundial de Saúde
SAMHSA	Abuso de substâncias e mental health services association
SARS-CoV-2	Coronavírus da síndrome respiratória aguda grave 2
SPSS	Statistical Package for Social Science
TCLE	Termo de Consentimento Livre e Esclarecido
